# ¿Where do migratory fish spawn in a neotropical Andean basin regulated by dams?

**DOI:** 10.1371/journal.pone.0291413

**Published:** 2023-10-19

**Authors:** Kelly Rivera-Coley, David Augusto Reynalte-Tataje, Víctor Atencio-Garcia, Omer Campo, Luz Jímenez-Segura

**Affiliations:** 1 Department of Biology, Ichthyology Group of the Universidad de Antioquia, Universidad de Antioquia, Medellín, Antioquia, Colombia; 2 Postgraduate Program of Environment and Sustainable Technologies (PPGATS), Fronteira Sul University (UFFS), Cerro Largo, RS, Brazil; 3 DCA/FMVZ/CINPIC, Universidad de Córdoba, Montería, Córdoba, Colombia; 4 Molecular Genetics Group, Universidad de Antioquia, Medellín, Antioquia, Colombia; DePaul University, UNITED STATES

## Abstract

Spawning sites play a key role in the reproduction of fish allowing populations to endure over time. The Nechí River is an important spawning area for potamodromous fish species where one of the threats is dam construction. In order to determine the importance of the Nechí River as a spawning site in the Magdalena River basin, sampling was conducted during the low-water-to-high-water season transition period between 2018 and 2019 at seven sampling sites. The average density of ichthyoplankton was 42.4 ind.10m-^3^ (SD = 7.1). Of the individuals in the post-larval stage, seven migratory species were identified, and two additional taxa were identified to genus; *Prochilodus magdalenae*, *Megaleporinus muyscorum*, and *Pseudoplatystoma magdaleniatum* presented the greatest density. At the temporal level, the greatest density of larvae of potamodromous species was observed in the first high-water season of 2019 with a total of 5.7 ind.10m^-3^(SD = 1.044), of which the most representative at the seasonal level were the Cauca River, Magdalena River, and Nechí River before it flows into the Cauca River. There were significant differences in the frequency of embryos and vitelline larvae of the potamodromous species in the interaction of the sampling sites and high-water seasons, as well as with the density of post-larvae. The average drift distance of the spawning areas is roughly 52.1 km. A positive association was found between the volume of turbined water and the presence of ichthyoplankton in the Porce River site, after discharge from the Porce III Hydroelectric Plant. The Nechí River is an important spawning site and there seems to be an association between the increase in ichthyoplankton densities and the distance to the dam (Porce III) as long as there are floodplains along the course of the river.

## Introduction

Due to the influence of anthropogenic activity, global biodiversity is decreasing at an alarming rate; the extinction rate is currently hundreds of times greater than the natural extinction rate [1, 2]. Fish have contributed to manufacturing and industry, technology, gastronomy, among others, for decades [3]. The main factors influencing a decreased diversity in freshwater fish worldwide include habitat change, extraction, introduction of non-native species, dam construction, water pollution, and climate change [2]. The construction of dams is considered the main cause of water ecosystem fragmentation and degradation, which significantly affects the abundance and diversity of the ichthyofauna. Dams flood various important environments for the life cycle of the fish species, particularly, potamodromous species, which migrate exclusively in fresh waters, where they spend most of the year in rivers or floodplains and migrate towards tributaries [4]. These dams flood their spawning, feeding and refuge areas. In addition, they homogenize the ecosystem and modify lateral connectivity and natural hydrodynamics [2, 5, 6]. These migratory species play an important role in the aquatic ecosystem, since they act as ecological drivers in the structure and functioning of the ecosystem, modulators of biogeochemical processes, and transporters of nutrients and energy [4, 7]. They help in the exchange of genes between populations [4]. In South America, hydroelectric development is advancing quickly, and most of the main basins are impacted by the presence of dams, thus, it is imperative to design and develop effective strategies that allow for the preservation of ichthyic diversity and the supply of fish for fishermen. For this, it is necessary to know the population dynamics of fish, their life cycle, and their spawning and growth habitats.

The Magdalena River basin is considered a hotspot for endemism in many groups [5]. This river is home to 233 freshwater fish species [5, 8], 97% of which partially adjust [9] to one of the life strategies proposed by Winemiller and Rose [10] and Vazzoler [11]. The most species-rich life strategy is the opportunistic, followed by the equilibrium strategy, and finally the seasonal o periodic strategy [12]. Seasonal species migrate along the main channel of the rivers [13], seeking other water systems (tributaries, streams, and floodplains) with the goal of finding adequate conditions to complete their life cycle [14–16] and breed [17]. In this Andean river that flows into the Caribbean Sea, there are at least 30 potamodromous fish species [12], most of which travel long distances [5] twice per year from low areas to high areas, reaching up to 1,300 mamsl [18].

The parts of the fluvial network where fish choose to migrate and spawn are in the main channel or in tributaries in portions of the channel with gentle slopes below 700 meters and near floodplains [19]. These species’ longitudinal upstream migration is known as *subienda y mitaca* (shoal) and occurs during the months in which the rivers reduce their flow rate, while longitudinal downstream migrations, known as *bajanza y rejarda*, occur when river flow increases during rainy season [20]. When the rainy season begins, the flow rate increases, resulting in the mating of migratory fish, which launches spawning and fertilization. Later, adults, eggs, embryos, and larvae (ichthyoplankton) drift downstream through the river’s main channel. During the downstream drift, embryos advance in their development, and with the high water levels and flooding of the river on its banks, the larvae enter marshes through streams [16, 19]. Most of these migratory species are the foundation of the country’s inland fisheries, where nearly 80% are economically important [21]. The most important species reported include bocachico *Prochilodus magdalenae*, jetudo *Ichthyoelephas longirostris*, dorada *Brycon moorei*, picuda *Salminus affinis*, arenca *Triportheus magdalenae*, mohíno *Megaleporinus muyscorum*, vizcaína *Curimata mivartii*, nicuro o barbudo *Pimelodus yuma*, capaz *Pimelodus grosskopfii*, bagre rayado *Pseudoplatystoma magdaleniatum*, and blanquillo *Sorubim cuspicaudus* [20].

The potamodromous fish in the Magdalena River spawn in the main channel in the middle basin and in the tributaries of the main course, principally rivers with low longitudinal slopes and near floodplains [12, 21, 22]. The most important tributaries for spawning are the Nechí (tributary of Cauca River), Sogamoso, Carare, and Opón [12, 19, 22]. The greatest spawning intensity occurs in the first high-water season of the year [12], which is preceded by upstream migration during the first seasonal baseflow [20]. These fish prefer to spawn at night [23], and lower water temperatures and increased concentration of sediment stimulate spawning [20, 24].

Many dams have been built along the Cauca River, a main tributary of the Magdalena River, in order to generate electric energy. Along the main course of the Cauca River, we find Salvajina (1,146 mamsl) built in 1985, and in 2014 the course of the river was changed to begin the construction of the Ituango Dam within the river’s main course (200 mamsl). In the Nechí River basin, a main tributary to the Cauca River, we find Riogrande II (2,270 mamsl), Guadalupe (2,057 mamsl), Porce II (700 mamsl), and Porce III (500 mamsl). This network of dams in the Cauca River currently generates nearly 1,960 MW (13.6% of the hydropower installed capacity in Colombia). In addition, the Ituango Dam is under construction and production is expected to increase to 4,360 MW when it begins to function.

The dams that generate electric energy in this basin have been identified as important disruptors of the reproductive cycle in these species, as they restrict migration [25] and they modify the hydrological regime that stimulates reproduction [26]. The Nechí River is an important location for spawning and development of potamodromous fish within the Magdalena River basin [19]. However, it is unclear whether the presence of dams within the Porce River (tributary of Nechí River) has an impact on how the potamodromous fish use the basin to migrate and spawn [27]. To make progress in this respect, we posed the following questions: Is the Nechí River still an important spawning location in the Magdalena River basin? Where do fish spawn within the Nechí River basin? Does spawning intensity maintain the same seasonal nature as reported in previous studies? Are the spawning locations particular to migratory species? Does the presence of Porce III Dam impact spawning intensity within the drainage network of the Nechí River? The aim of this study is to determine the importance of the Nechí River as a location for spawning and initial development of potamodromous fish species in the Magdalena River basin. This will contribute to the planning of the basin and to the management required by the power generating reservoirs for the protection of these areas and these species.

## Materials and methods

### Ethics statement

This study was conducted with the recommendations and approval of the Ethics Committee for Animal Experimentation from the Universidad de Antioquia (CEEA). The protocol was reviewed and approved in November 14 of 2017 by CEEA and the investigation on December 7 of 2017. Besides, the specimen collection was conducted with the approval of the Ministry of Environment, granting permit through resolution 0524 of May 27, 2014.

### Study area

The Nechí River begins in Llanos de Cuivá (Yarumal, Antioquia, Colombia) at 2,730 mamsl, is 252 km long, and flows into the Cauca River at 30 mamsl. The basin is formed by tributaries with narrow valleys and steep slopes; after receiving the Porce River, its main tributary, the river slope reduces and forms a vast alluvial valley with marshes in its riverbanks connected by sinuous streams. Rainfall in the basin ranges from 1,000 to 4,000 mm.year^-1^ and presents a bimodal pattern as follows: April through June, and September through October. The average flow rate is 830 m^3^.s^-1^ with two low-water seasons and two-high water seasons. For this region, the threats to migratory species come from mining, agriculture, livestock, fishing, logging and hydroelectric activities.

### Experimental design

**Fig 1:**
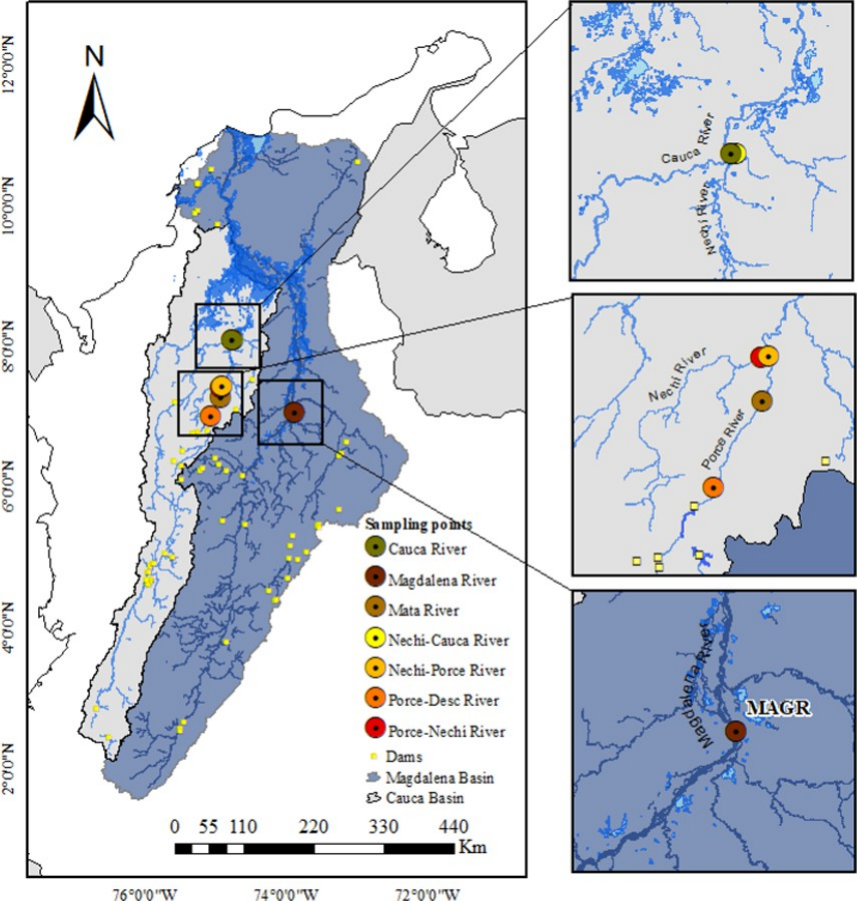
Sampling sites of ichthyoplankton in the Nechí, Cauca, and Magdalena Rivers. The small yellow squares indicate the location of dams. Abbreviation MAGR: Magdalena River. The shapefile of basins was obtained from IGAC (https://geoportal.igac.gov.co/). All other products were produced by the authors and are copyright-free.

During three transition periods between low-water and high-water seasons in the basin (September 2018, April 2019, September 2019), samples were taken for 15 consecutive days in seven locations along the Nechí, Cauca, and Magdalena River basins (Fig 1). Before taking samples, the position of the channel was located within the course to measure the depths in the perpendicular transects in each location. Samples were simultaneously taken at 08:00 h in each location in the channel of the section of the river.

The ichthyoplankton samples were obtained using a conical-cylindrical plankton net (400 μm mesh, 0.38 m mouth diameter) immersed at 0.5 m below the water surface and placed horizontally to filter against the flow [20]. The volume filtered water was estimated using a flowmeter fixed into the net mouth.

Two replicates of ichthyoplankton were taken at every site on each day. One of the samples was fixed in 4% formaldehyde and the other in 96% alcohol. The ichthyoplankton collected was preserved in Transeau solution or 96% alcohol and labeled with date and collection site.

### Ichthyoplankton identification

Using a stereo microscope (Leica) with 10X to 80X magnification, the ichthyoplankton was separated according to developmental stages as follows: embryo, vitelline larva, and post-larva (preflexion, flexion, and postflexion) [17, 28, 29]. The dichotomous key of species of the Magdalena River basin [30] was used for the taxonomic identification of the larvae of migratory species; however, in cases where a specific identification was not possible, they were grouped according to taxonomic order, such as Characiformes (Chara 8, Chara 9, Chara 25, Chara 33) and Siluriformes (Sil 12) [20]. Individuals whose characters did not fit any known taxa descriptions were assigned to the “Undetermined (NN)” category. For taxon identification of individuals in embryonic and vitelline larval stages, DNA barcoding was used [31]. In a fragment of the cytochrome oxidase type I (cox1) mitochondrial gene was amplified and sequenced using the primers FishF1 (5’-TCAACCAACCACAAAGACATTGGCAC-3’) and FishR1 (5’TAGACTTCTGGGTGGCCAAAGAATCA-3’) proposed by Ward et al. [32].

Dna extraction was performed by grouping several individuals in embryonic and larval viteline stage. Amplification was done by conventional PCR, using specific primers for each species, which were designed in the ichthyology laboratory of the University of Antioquia S1 Table. Each PCR mix contained 2.0 μL of DNA, 1X Taq Buffer, 2.5 mM MgCl2, 0.2mM dNTPs, 0.2 μM of each primer, 0.060 U of Taq Polymerase (Thermo Scientific EP0406) in a final volume of 30 μL. The amplification started at 94°C for 5 min, followed by 34 cycles of 94°C for 35 s, 56°C for 45 s, 72°C for 1min, and a final step at 72°C for 10min. The amplicons were tested by 3.5% agarose gel electrophoresis, determining each species by size in base pairs, so that a priori they were grouped into different groups for identification. This method made it possible to identify the presence of the species, but not quantify theirabundance.

### Frequency of occurrence and ichthyoplankton density

To determine the importance of the Nechí River as a spawning site for potamodromous species in the Magdalena River basin, frequency of occurrence charts of spawning potamodromous species were made based on the number of times each species was present in each of the samples at every site with respect to total samples in each sampling period and year. Ichthyoplankton density (ind.10m-3) was estimated from the sample abundance and the volume of water filtered [17, 33]. R version 3.6.3 was used to conduct a non-parametric test (Kruskal-Wallis) to analyze the differences between the frequency of occurrence of potamodromous species in embryonic and vitelline larval stages, as well as the density of post-larvae amongst the various collection sites along this river [34]. A Wilcoxon Mann-Whitney test was also performed to compare the ichthyoplankton densities between the Nechí River collection site (before flowing into the Cauca River) to determine if it is a spawning area and the site in the Magdalena River next to the city of Barrancabermeja, which we know a priori to be a spawning area. In all cases, the variables analyzed were subject to assumptions of homogeneity (Shapiro Wilk test).

### Spawning locations

To identify the location of the spawning sites within the Nechí River basin, a spatial and temporal distribution chart of spawning was created based on the distribution of the frequency of embryos and vitelline larvae as well as the density of post-larvae at each sampling site and high-water season.

To determine the geographical location of the spawning site, we estimated the ichthyoplankton drift distance. This distance was estimated based on the post-fertilization incubation time of the embryos and larvae collected, keeping in mind the average speed of the river at the time of sampling, which was calculated using a flowmeter (General Oceanics, 2030R). The incubation time for each period and development phase for migratory species was obtained from information available in the literature [35–38].

## Results

### Density by early developmental stage

**Fig 2:**
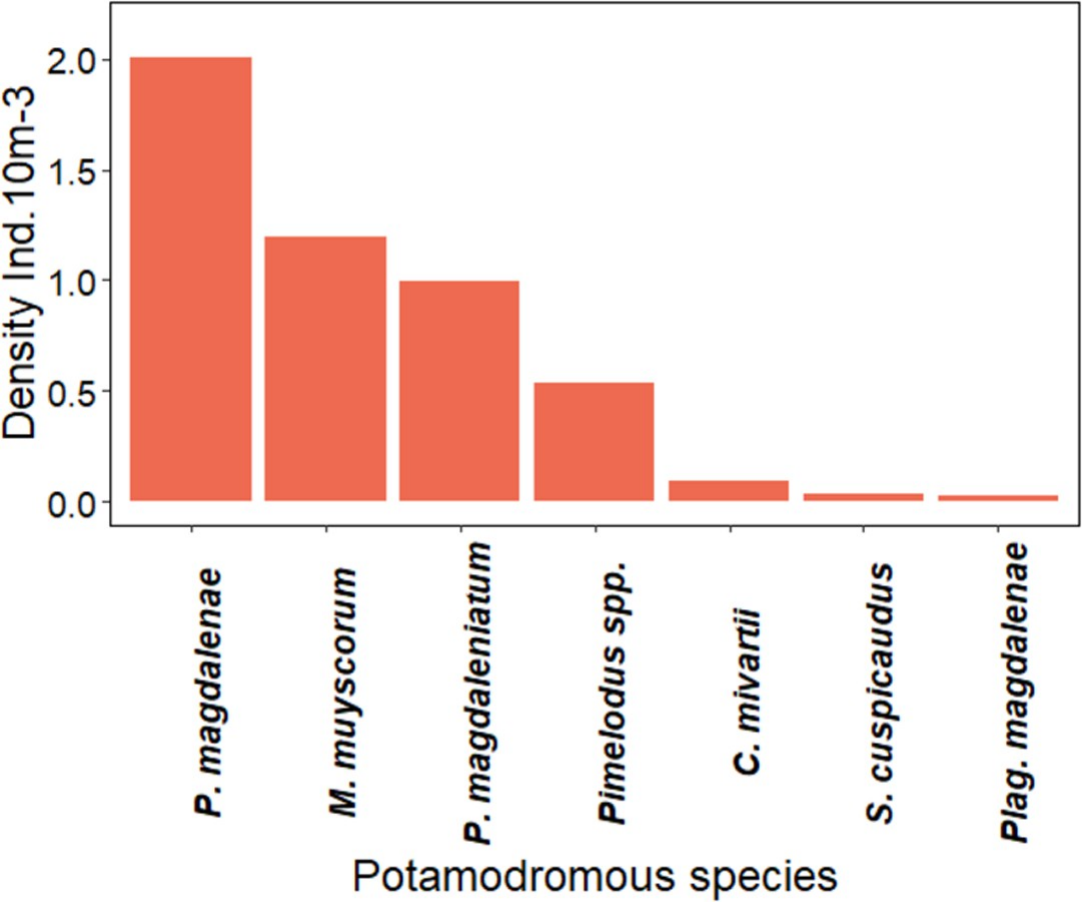
Density of potamodromous species. Species in larval stage during the high-water season of September of 2018, and April and September of 2019 in the Magdalena River basin.

**Fig 3:**
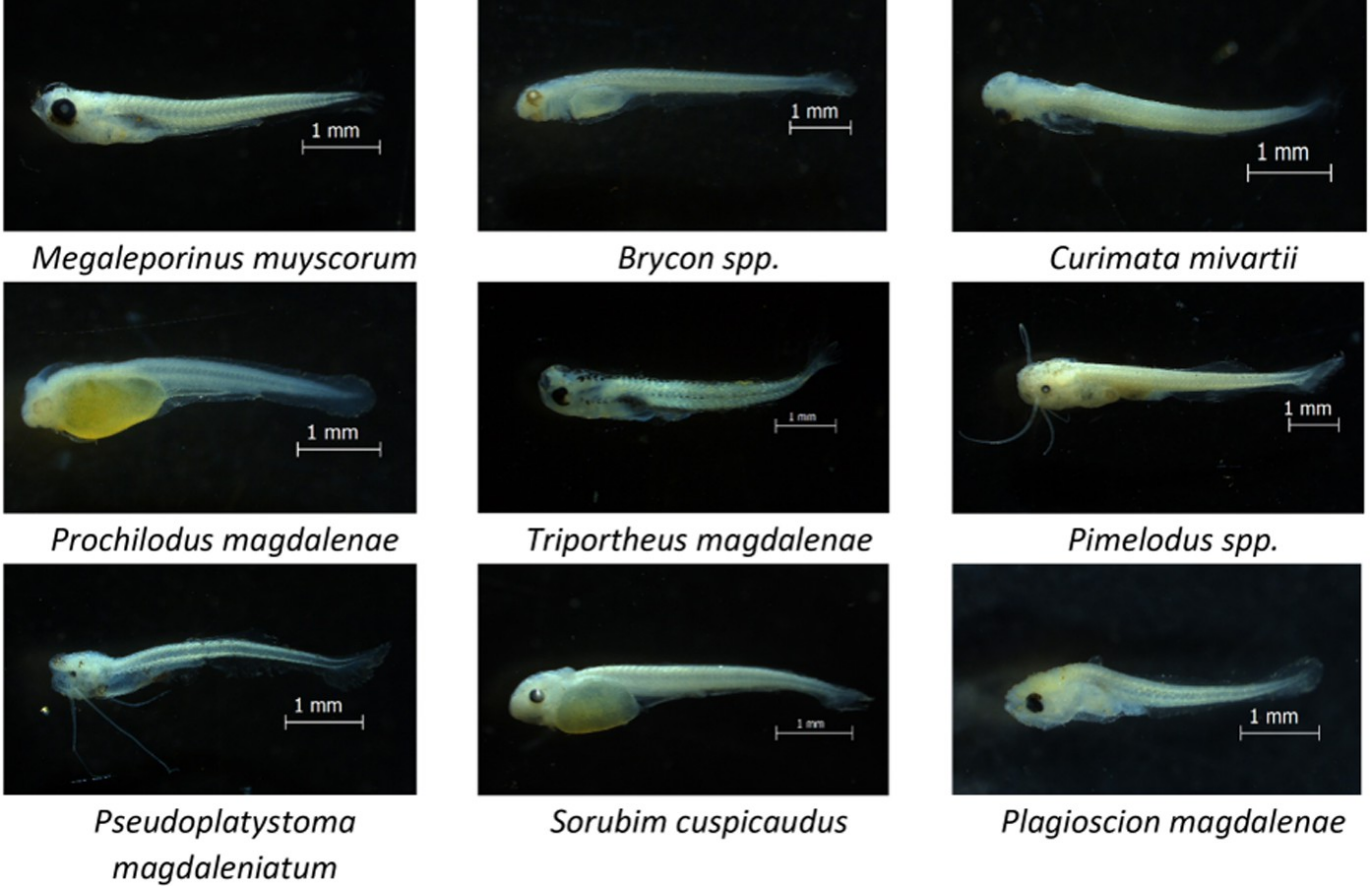
Potamodromous species in the Magdalena River basin.

Twenty taxa were identified in the ichthyoplankton collected, of which 70% were potamodromous; in addition, it was found that ten species and one genus were in embryonic or vitelline larval stages. For the post-larvae (preflexion, flexion, and postflexion) collected, seven migratory species were recorded (*P*. *magdalenae*, *M*. *muyscorum*, *C*. *mivartii*, *S*. *cuspicaudus*, *P*. *magdaleniatum*, *T*. *magdalenae* and *Plagioscion magdalenae*) and two species identified to genus (*Pimelodus* spp. and *Brycon* spp.) (Fig 2). The average density of ichthyoplankton was of 42.4 ± 7.1 ind.10m-^3^ (91.6% larvae and 8.4% embryos) where *P*. *magdalenae* (2.0 ind.10m^-3^), *M*. *muyscorum* (1.2 ind.10m^-3^), and *P*. *magdaleniatum* (1.0 ind.10m^-3^) were the most abundant species (Figs 2 and 3 and Table 1 and S2 Table).

**Table 1:** Density and frequency of early developmental stages of the fish species.

Especie	Embryo	Larvae	Frequency (%)		
		Ind.10m^-3^	2018	2019–1	2019–2
Megaleporinus muyscorum*	X	1.2	9.5	18.8	19.0
Brycon spp.*		0.0	0.0	2.1	0.0
*Cynopotamus magdalenae*	X	0.0	0.0	0.0	0.0
Curimata mivartii*	X	0.1	4.8	8.3	4.8
*Cyphocharax magdalenae*	X	0.0	0.0	0.0	0.0
Prochilodus magdalenae*	X	2.0	4.8	11.5	9.5
Triportheus magdalenae*	X	0.0	0.0	0.0	1.0
Chara 8		0.0	1.0	0.0	0.0
Chara 9		0.0	0.0	1.0	0.0
Chara 25		0.0	0.0	0.0	0.0
Chara 33		0.0	1.0	0.0	0.0
Chara NN		31.9	41.9	49.0	44.8
Pimelodus spp.*	X	0.5	8.6	18.8	15.2
Pimelodus grosskopfii*	X	0.0	0.0	0.0	0.0
Pimelodus yuma*	X	0.0	0.0	0.0	0.0
Pseudopimelodus spp.*	X	0.0	0.0	0.0	0.0
Pseudoplatystoma magdaleniatum*	X	1.0	12.4	15.6	14.3
*Sorubim cuspicaudus*		0.0	4.8	1.0	3.8
Sil 12		0.0	1.9	6.3	8.6
Sil NN		0.6	3.8	21.9	20.0
Plagioscion magdalenae*		0.0	1.0	4.2	1.0
Gimnotiformes		0.0	4.8	3.1	1.0
LV15		0.0	1.0	0.0	0.0
Others(SI)		1.2	5.7	15.6	25.7

*potamodromous species. The X’s indicate the presence of the species in embryo stage and the frequency in relation to larval stage, therefore those species with frequency 0.0 in all samples indicate that no larval stage individuals were collected. LV15: morpho 15 in vitelline larval stage. Others (SI): individuals not identified.

**Fig 4:**
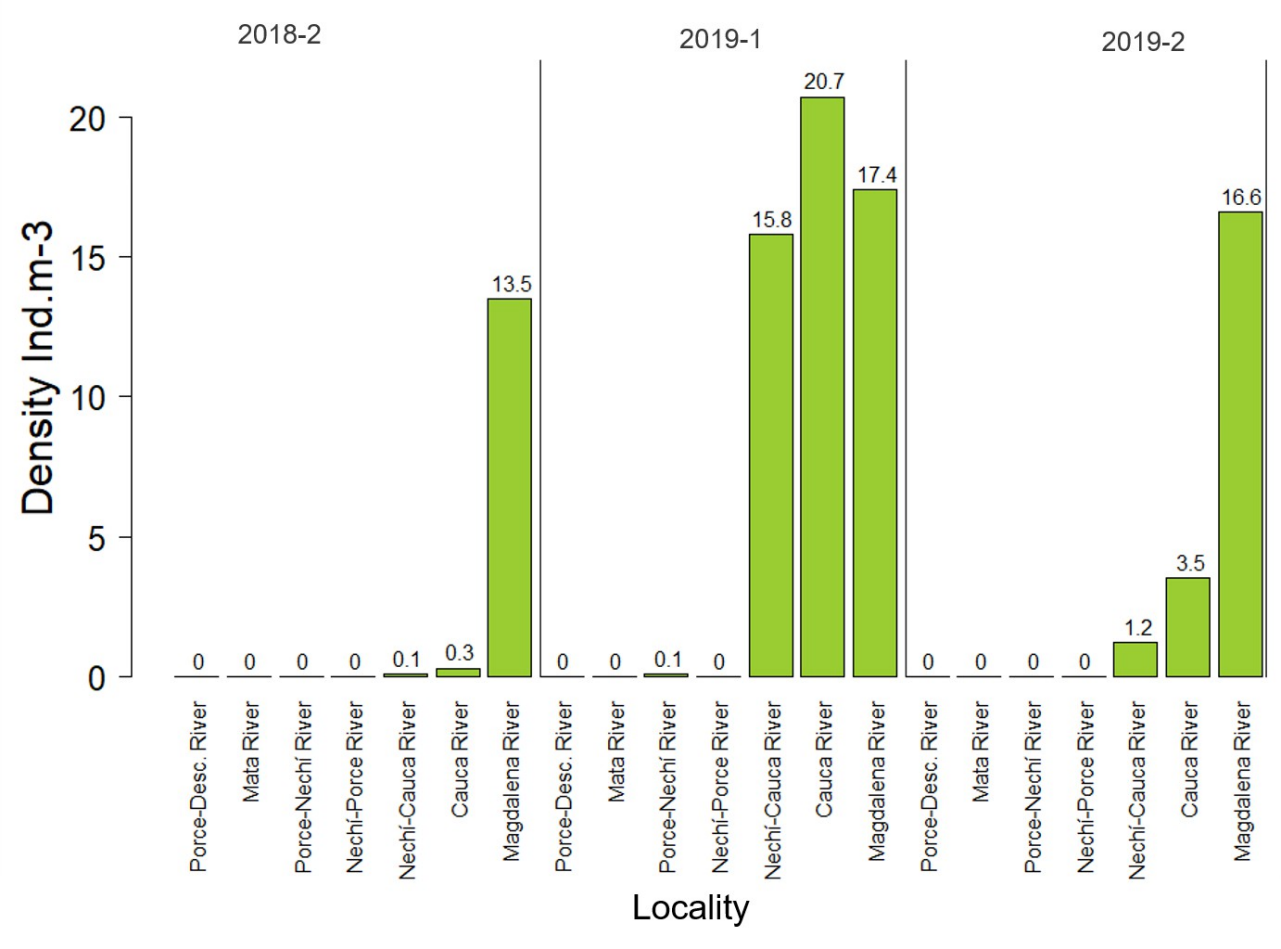
Distribution of the larval density of potamodromous species at each location, and high water level of the Magdalena River basin.

The highest density of larvae of potamodromous species was recorded during the first high-water season of 2019 (5.7 ind.10m^-3^(SD = 1.044)) in the Cauca River (20.7 ind.10m^-3^), Magdalena River (17.4 ind.10m^-3^), and Nechí River before the place where it flows into the Cauca River (15.8 ind.10m^-3^) (Fig 4). The highest larvae densities were also recorded at these same sites in the second high-water season of 2019 (1.8 ind.10m^-3^) and the second high-water season of 2018 (1.2 ind.10m^-3^) (Fig 4).

### Frequency

**Fig 5:**
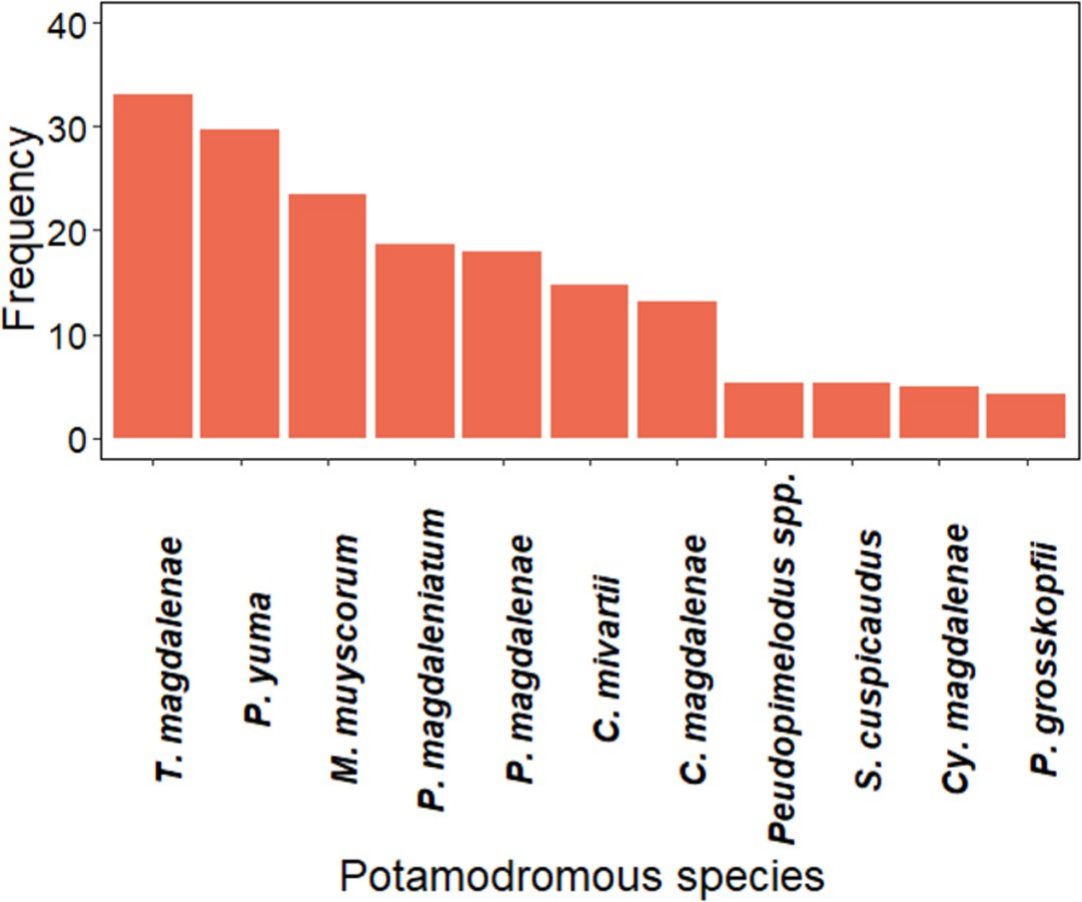
Occurrence of potamodromous species in embryonic and vitelline larval stages in the Magdalena River basin.

Ten species and one species identified to genus were recorded during this study in terms of frequency and according to embryo and vitelline larval stages of potamodromous species. The most frequent potamodromous species were arenca *T*. *magdalenae* (33%), nicuro *Pimelodus yuma*. (29.7%), and Comelón *M*. *muyscorum*, (24%) (Fig 5).

**Fig 6:**
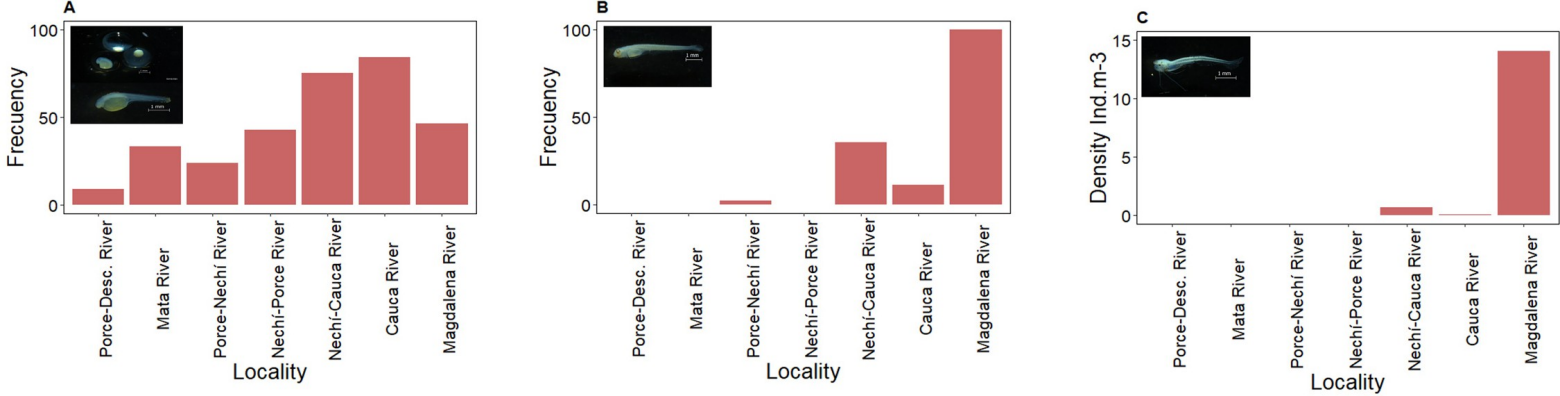
Distribution of the frequency and density of embryos and larvae of potamodromous species at the various locations along the Magdalena River basin. A) frequency of embryos and vitelline larvae; B) frequency of post-larvae; C) density of post-larvae.

Significant differences were found in the frequency of embryos and vitelline larvae of potamodromous species amongst the various locations (K-W:62.346; p:0.001). Significant differences were also found with respect to the density of post-larvae (K-W:233.28, p<0.001) (Fig 6).

**Fig 7:**
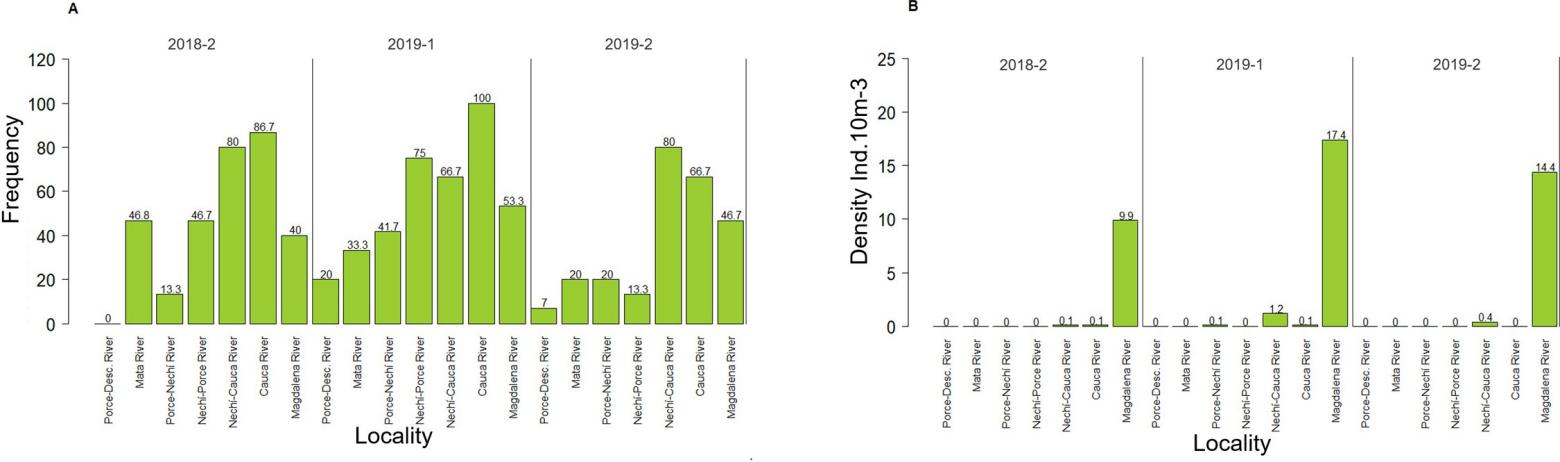
Distribution of the frequency and density of the ichthyoplankton at each location and high-water season of the Magdalena River basin. A) frequency of embryos and vitelline larvae; B) density of post-larvae.

Significant differences were found in the frequency of embryos and vitelline larvae of potamodromous species in the interaction of the sampling locations and high-water seasons (K-W: 98.158; p:0.001); significant differences were also recorded with respect to the density of post-larvae in the interaction of sampling sites and high-water seasons (K-W: 243.92; p:0.001) (Fig 7). The density of migratory species also differed significantly between the Nechí-Cauca River and Magdalena River (W: 1988; p:1.57e-15).

### Distribution of migratory species during early developmental stage

**Fig 8:**
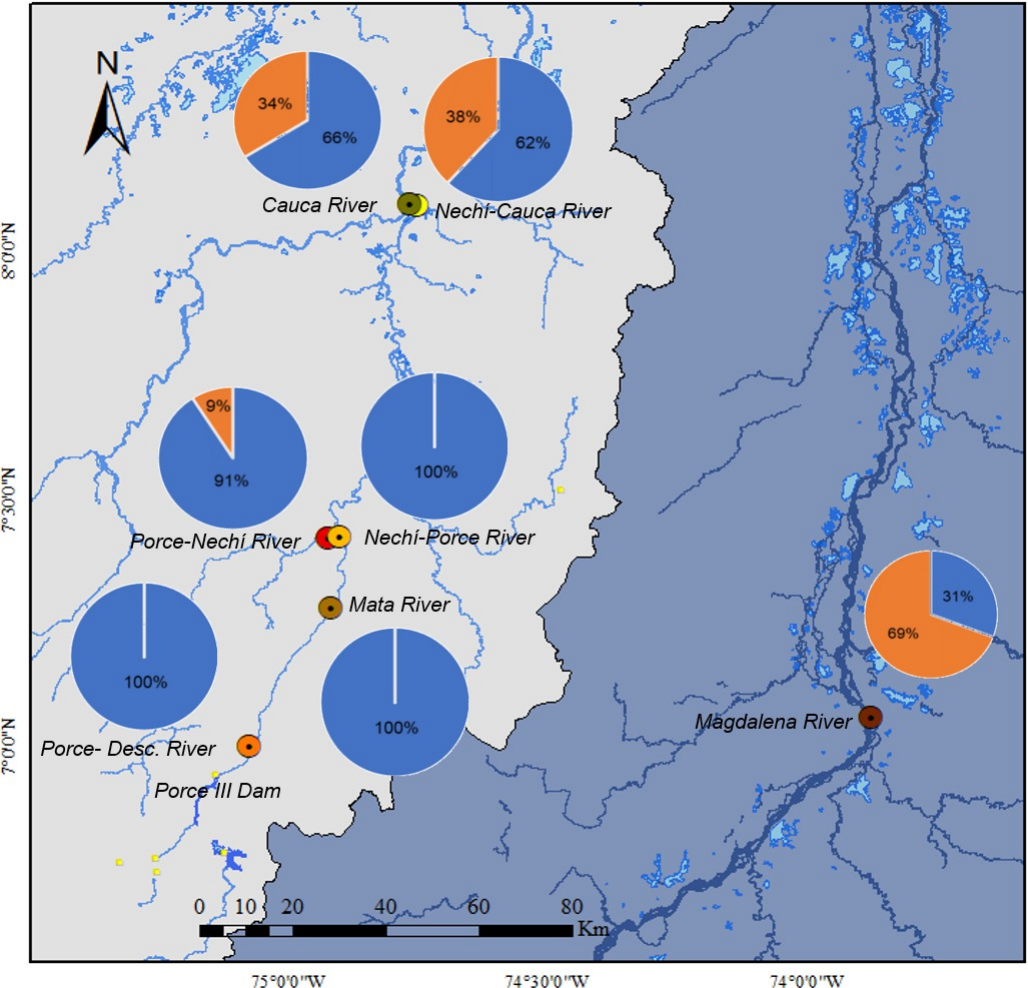
Distribution of the frequency of embryos and larvae of potamodromous species at sampling sites. Blue represents embryo and larval stage individuals, while orange represents post-larvae. The shapefile of the basins was obtained from IGAC (https://geoportal.igac.gov.co/). All other products were produced by the authors and are copyright-free.

The potamodromous species in their various developmental stages (embryos and vitelline larvae) were recorded at each location (Porce River-discharge, Mata River, and Nechí-Porce River) while the post-larvae were recorded in a mostly in the Magdalena River location (Fig 8).

### Spawning sites

It was estimated that the spawning sites of the potamodromous species recorded in this study are found at an average distance of 52.1 km, and an interval between 8.5 km (Nechí River) and 77.4 km (Cauca River) from the sampling sites. In Characiformes, an average drift distance was estimated at 74.7 km to the collection site, oscillating from 8.4 km (embryonic stage individuals) and 168.4 km (vitelline larvae). On the other hand, for Siluriformes, minimum and maximum distances recorded were 9.1 km and 138.4 km, respectively, and an average drift distance of 108 km. These distances were recorded by individuals in the embryonic and vitelline larval stages (Table 2).

**Table 2:** Minimum and maximum estimated distances from spawning locations to sampling sites of potamodromous species in the Magdalena River basin.

Station	Distance Km					
	General		Characiformes		Siluriformes	
	Minimum	Maximum	Minimum	Maximum	Minimum	Maximum
Porce-Descarga River	10.3	66.3	8.4	40.1	9.1	46.7
Mata River	15.3	68.8	26.3	81.5	21.2	129.4
Porce-Nechí River	8.6	71.8	24.2	94.9	26.2	234.3
Nechí-Porce River	8.5	64.8	13.7	48.2	10.6	125.6
Nechí-Cauca River	23.1	65.4	22.8	142.4	24.5	118.5
Cauca River	17.5	77.4	33.4	168.4	39.7	138.4
Magdalena River	18.9	52.6	23.2	99.3	25.1	119.4

## Discussion

Our study confirms that migratory fish spawn in various locations along the Magdalena-Cauca basin, using the main channels of these rivers as well as their main tributaries. In addition, the presence of 14 potamodromous species (of the 30 reported for the basin) was reported in the ichthyoplankton, which highlights the importance of the region for the life cycle of these organisms, despite the presence of dams.

In neotropical basins, fish spawning occurs in the main river course [39–41], in tributaries [19, 22, 42], and at their mouths [39]. In the upper Paraná River, one of the most studied regions of the Neotropics, migratory species spawn at the headwaters of the tributaries of large rivers [43, 44], whereas, in the Uruguay River, they species use the confluence of the rivers [45] or the main river course [46] to fulfill this reproductive event.

In the Magdalena-Cauca basin, it was observed that tributaries, such as Porce and Mata Rivers, located in the upper parts of the study area at an altitude of 87 to 340 masl, presented individuals in embryonic and vitelline larval stages, as in the Nechí River before its confluence with Porce River. Thus, the presence of embryos and vitelline larvae suggests proximity to a spawning site [47]. When considering that the embryos of potamodromous fish generally hatch 14–20 hours post-fertilization (depending on the water temperature), this suggests that when embryos are being collected, the spawning site is approximately 20 hours away [35–38] depending on the species, developmental stage, and water speed upstream from the sampling site.

On the other hand, the distribution of post-larvae in the study area increased in terms of individuals at sites near the floodplains, which are found at an altitude of 30 to 70 mamsl in the Magdalena, Cauca, and Nechí Rivers. The floodplains are ecosystems with an abundance of vegetation cover [48], high biological productivity [49–52], and where larvae find food [52–54] and refuge to continue their development [52, 55]. Potamodromous fish migrate in the adult stage towards nearby rivers with connectivity to the areas of growth, thus allowing the entry of post-larvae to the floodplains [22]; this explains the presence of post-larvae near floodplains and highlights the importance of these systems for species growth.

Spawning sites play an important role in the reproduction of fish as they allow populations to endure over time [41]. In the Magdalena-Cauca basin, these spawning sites are found in the main course and/or tributaries in portions of the channel with gentle slopes and near floodplains [19]. The presence of potamodromous species in early stages within tributaries, such as Porce River and Mata River, and in the main channel of the Nechí River, confirms that this subbasin remains an important spawning site, which had been suggested by a study on the high-water seasons of 2013–2014, where the Cesar, San Jorge, Magdalena (the portion near Barrancabermeja and Honda), Nechí, Sogamoso, Carare, and Opón Rivers are mentioned as the most important spawning areas for these species [12, 19, 22]. Another study reported that the spawning sites of the Cauca River are in the area between Puerto Valdivia and Mantequera (Pinillos), as well as in its tributaries (Tarazá and Nechí Rivers) [56].

In this study, the species recorded in the rivers being observed sustain the fisheries resource of the Magdalena-Cauca basin. These species include *P*. *magdalenae*, *P*. *magdalenitum*, *S*. *cuspicadus*, *M*. *muyscorum*, amongst others [21, 57, 58], which coincides with the densities recorded in the ichthyoplankton samples taken along the entire basin [16, 20, 22]. The density of ichthyoplankton recorded in the samples taken is mainly due to the contribution of the collection performed in 2019–1, and may be associated with the migratory event of the subienda (shoal), a more intense dry season, the low water quality in the floodplains, or a greater number of migrating individuals.These species reproduce in high-water seasons under particular conditions; some species even spawn before the high-water seasons (e.g., *S*. *cuspicaudus*) [20]. The species that spawn at the beginning of the high-water seasons prior to inundation of the riverbanks are known as “risk-takers”, whereas the species that spawn during the high-water seasons, following inundation of the riverbanks are known as “care-takers” [20, 59]. Therefore, *P*. *magdalenae*, *M*. *muyscorum*, *C*. *mivartii*, *P*. *magdaleniatum*, and *Pimelodus spp*. seem to adopt the careful strategy while *S*. *cuspicaudus* adopts the riskier one.

The spawning of *P*. *magdalenae*, *M*. *muyscorum*, *C*. *mivartii*, *P*. *magdaleniatum*, *S*.*cuspicaudus*, *S*. *affinis* and *Pimelodus spp*. concentrates in the tributaries and in the main channel of the Magdalena River [19, 20, 22] (S3 Table). In neotropical fish there is a synchronization between the hydrological cycle and reproduction [11, 60–63], especially in migratory fish that release many eggs in the water column without parental care [63, 64].

Some authors mention that the intensity of the drought could impact the number of migrating individuals, and hence, the amount of spawning adults [16, 18, 65, 66]. The duration of the hydrological cycles are determining in spawning and recruitment of species since it has been reported that the absence, delay, and magnitude of flooding has repercussions in reproduction (migration, spawning, recruitment) [63, 67, 68]. For example, if no flooding occurs, the eggs and larvae that drift in the water column would not enter the areas of growth and refuge, and thus, would remain in the main river channel, leaving them, in most cases, without the necessary feeding and refuge conditions for their development [63].

On the other hand, it has been detected that the El Niño-La Niña cycle favors the recruitment of fish since spawning is intense following the long low-water season that characterizes El Niño, and, with the arrival of La Niña, flooding of large areas over several months favors the survival of larvae and the recruitment of spawning fish populations [63]. However, it is important to note an interesting phenomenon in 2019–1 involving the elevated density of ichthyoplankton in the Cauca and Nechí Rivers, which could have been due to a blockage in the tunnel of the Ituango Hydroelectric plant, resulting in a decrease in water level downstream in the main course of the river and in the tributaries and floodplains; this was followed by intense spawning of migratory species during rainy season, which may have been a survival method for these species, given that, when they found themselves in adverse conditions, they reproduced vehemently and thus maintained the species over time.

Potamodromous fish perform longitudinal migration upstream for reproduction from the floodplains in the lower sections toward the main course of rivers and tributaries during the low-water season. Later, with the high water levels, they migrate downstream to return to the floodplains accompanied by their offspring [20, 22]. The distribution at the spatial level of these life stages could be indicating the location of the spawning sites as well as the growth and refuge areas for migratory species.

Hydroelectric plants generate changes in the frequency, intensity, and pulses of water flow, causing stress in all life stages of fish that live downstream [69]. One of the negative impacts of hydroelectric plants is the disorientation of the species during spawning season due to hydropeaking, which consists in the discontinuous release of turbined water to meet peak daily electricity demands for these plants, causing fluctuations in the water flow and affecting the environmental stimuli necessary for the reproduction of migratory species [70]. In the Porce River, especially in the discharge area, embryos of potamodromous species were found, suggesting it is a spawning area. However, the conditions of the flow regime have been modified by the Porce III hydroelectric plant, affecting the final environmental stimuli for reproduction. The presence of these species could be due the fact that they possibly were unable to take an alternate route to migrate and spawn. Nonetheless, previous studies have shown that species like *Brycon rubricauda*, *I*. *longirostris*, *M*. *muyscorum*, and *P*. *magdalenae* spawn downstream from the Porce III dam, especially during operation [12].

Furthermore, the presence of embryos in the Mata River could be due to the fact that these species took this tributary as an alternate route, given the proximity to the Porce III dam, a site where downstream spawning of potamodromous species has been evidenced; however, there are no data prior to the construction of the dam to confirm this hypothesis. Although there are no historical data, the high amount of captures of eggs or embryos in tributaries near a dam could indicate that they are an alternate migratory route [45, 71]. It is possible that after dam construction and river fragmentation, fish that migrated in the main channel and tributaries may move laterally in tributaries with characteristics of lotic systems [72]. Further study is warranted in order to confirm this hypothesis in the Porce and Mata Rivers.

Lastly, we can conclude that potamodromous fish use the channels of rivers and tributaries to spawn, and that its intensity depends on the duration of the low-water season and the number of migrating adults. In addition, given the proximity of the floodplains due to the low slope and the connectivity that remains amongst the various water systems (main channel, tributary, stream, floodplain), the Nechí River continues to be an important area for potamodromous fish reproduction, which makes viable the survival of migratory fish offspring, hence, the main channel and tributaries allow for the development of the individuals that drift. Furthermore, it should be noted that for the nation’s hydropower planning it is important to consider the tributaries that remain unregulated in the hydroelectric development that have similar or greater water levels than regulated rivers and that converge downstream from these rivers, since these unregulated rivers could be an alternate migratory route and may alleviate the conditions generated by the daily changes in water level caused by these hydroelectric plants [73].

## Supporting information

S1 Table Primers potamodromous species. (XLSX)

S2 Table List of coordinates of the different species collected in larval stage. (XLSX)

S3 Table Potamodromous species spawning sites. (XLSX)
